# DysRegSig: an R package for identifying gene dysregulations and building mechanistic signatures in cancer

**DOI:** 10.1093/bioinformatics/btaa688

**Published:** 2020-07-27

**Authors:** Quanxue Li, Wentao Dai, Jixiang Liu, Qingqing Sang, Yi-Xue Li, Yuan-Yuan Li

**Affiliations:** School of Biotechnology, East China University of Science and Technology, Shanghai 200237, China; Shanghai Center for Bioinformation Technology, Shanghai 201203, China; Shanghai Center for Bioinformation Technology, Shanghai 201203, China; Department of Surgery, Shanghai Key Laboratory of Gastric Neoplasms, Shanghai Institute of Digestive Surgery, Ruijin Hospital, Shanghai Jiao Tong University School of Medicine, Shanghai 200020, China; Shanghai Engineering Research Center of Pharmaceutical Translation, Shanghai Industrial Technology Institute, Shanghai 201203, China; Shanghai Center for Bioinformation Technology, Shanghai 201203, China; Shanghai Engineering Research Center of Pharmaceutical Translation, Shanghai Industrial Technology Institute, Shanghai 201203, China; Department of Surgery, Shanghai Key Laboratory of Gastric Neoplasms, Shanghai Institute of Digestive Surgery, Ruijin Hospital, Shanghai Jiao Tong University School of Medicine, Shanghai 200020, China; School of Biotechnology, East China University of Science and Technology, Shanghai 200237, China; Shanghai Center for Bioinformation Technology, Shanghai 201203, China; Shanghai Engineering Research Center of Pharmaceutical Translation, Shanghai Industrial Technology Institute, Shanghai 201203, China; CAS Key Laboratory of Computational Biology, CAS-MPG Partner Institute for Computational Biology, Shanghai Institutes for Biological Sciences, Chinese Academy of Sciences, Shanghai 200031, China; Shanghai Center for Bioinformation Technology, Shanghai 201203, China; Shanghai Engineering Research Center of Pharmaceutical Translation, Shanghai Industrial Technology Institute, Shanghai 201203, China

## Abstract

**Summary:**

Dysfunctional regulations of gene expression programs relevant to fundamental cell processes can drive carcinogenesis. Therefore, systematically identifying dysregulation events is an effective path for understanding carcinogenesis and provides insightful clues to build predictive signatures with mechanistic interpretability for cancer precision medicine. Here, we implemented a machine learning-based gene dysregulation analysis framework in an R package, DysRegSig, which is capable of exploring gene dysregulations from high-dimensional data and building mechanistic signature based on gene dysregulations. DysRegSig can serve as an easy-to-use tool to facilitate gene dysregulation analysis and follow-up analysis.

**Availability and implementation:**

The source code and user’s guide of DysRegSig are freely available at Github: https://github.com/SCBIT-YYLab/DysRegSig.

**Supplementary information:**

Supplementary data are available at *Bioinformatics* online.

## 1 Introduction

Tumorigenesis is believed to be triggered by a series of events such as DNA mutation, chromosomal variation, aberrant epigenetic modification and further driven by dysfunctional regulation of gene expression programs (Hanahan and Weinberg, 2011; Lee and Young, 2013). Gene dysregulation has been suggested as a hallmark of cancer (Bradner *et al.*, 2017). The phenotypic heterogeneity of tumors, like drug response, metastasis and survival time, could thus be ascribed, at least in part, to gene dysregulations. In this sense, investigating transcriptional dysregulations helps to understand the molecular mechanisms underlying phenotypic changes and promotes the implementation of cancer precision medicine. Specifically, gene dysregulation analysis has the potential to provide functionally relevant seeds for building predictive signatures with both predictive power and explanatory power, which to some extent address the issue that most of current efforts to build signatures for predicting prognosis, and therapeutic benefits are focusing on predictive accuracy rather than on mechanistic interpretability (Robinson *et al.*, 2013).

About one decade ago, differential correlation analysis (DCA) began to emerge as a mechanism-driven strategy, representing the first steps towards elucidating gene dysregulations (de la Fuente, 2010). However, without fully incorporating transcriptional regulation rules, DCA-based methods inevitably include too much noise (Singh *et al.*, 2018). To enhance the performance of gene dysregulation analysis, we proposed a machine learning-based framework in a companion paper (Li *et al.*, submitted to *JMCB*, accepted), which is capable of robustly exploring gene dysregulations from high-dimensional expression data with cooperativity and synergy between regulators, and several other transcriptional regulation rules are taken into consideration. Here, we report DysRegSig, an R package that serves as an easy-to-use tool to facilitate gene dysregulation analysis and mechanistic signature construction for cancer precision medicine.

## 2 The design of DysRegSig

**Fig. 1.**
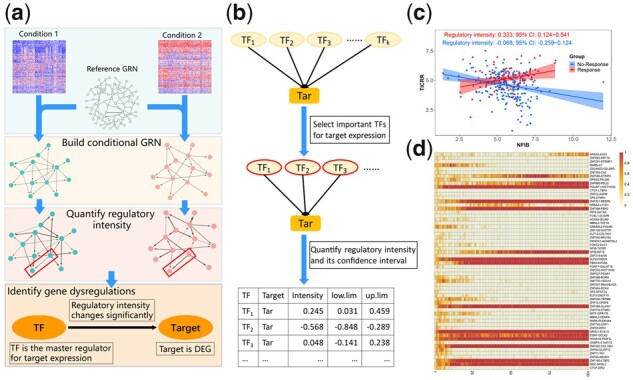
The framework of DysRegSig. (**a**) The pipeline for identifying gene dysregulations. (**b**) The details of the construction of conditional GRNs and the quantification of regulatory intensities. (**c**) An example of gene dysregulation. *X*-axis denotes TF’s expression and y-axis denotes target’s expression. The regression lines and confidence interval shadows were calculated by single variable regression. (**d**) The identification of gene dysregulations that is robustly associated with a specific phenotype. The frequency of each dysregulation in top ten ‘individuals’ at each iteration of genetic algorithm is indicated by color

Figure 1 describes the framework of DysRegSig. Figure 1a gives an overview of the pipeline for identifying gene dysregulations. Given expression data under two comparative conditions and a reference gene regulatory network (GRN), DysRegSig first builds conditional GRNs with feature selection algorithm such as Boruta (Kursa and Rudnicki, 2010) that is able to consider cooperativity and synergy between TFs and robustly cope with high-dimensional data, then quantifies regulatory intensities of every regulation relationships and their confidence intervals with de-biased LASSO (Javanmard and Montanari, 2014), and eventually identifies gene dysregulations by integrating three standards including differential regulation, differential expression of target and the consistency between differential regulation and differential expression (Li *et al.*, 2017) (Fig. 1a and b). Figure 1c provides an example of a gene dysregulation. Benefited from the above design, the gene dysregulation analysis pipeline could robustly process high-dimensional expression data with cooperativity and synergy between regulators, and several other transcriptional regulation rules are taken into consideration.

Based on the identified gene dysregulations, DysRegSig made further selections with genetic algorithm, focusing on those robustly associated with a specific phenotype, such as prognosis and drug response (Fig. 1d). These dysregulations could be used as mechanistic signatures for cancer precision medicine. Besides, DysRegSig offers DCA tools including *DiffCor* and *DiffCor++*, which combines traditional DCA, differential expression analysis, and the estimation of the consistency between differential coexpression and differential expression. Tools for ranking dysregulations and TFs, *RankTF* and *RankDysReg*, are also provided. More details of DysRegSig could be found in Supplementary File.

## 3 Case study

We adopted dataset IMvigor210CoreBiologies to demonstrate the practicability and performance of DysRegSig, which contains expression data from patients with metastatic urothelial cancer and matched drug response data of a PDL1 inhibitor (Mariathasan *et al.*, 2018). DysRegSig identified 295 gene dysregulations between response group (*n* = 68) and non-response group (*n* = 230) (Supplementary Table S1). Among the top ten TFs, eight are cancer-related genes (Supplementary Table S2, Supplementary Fig. S1). The identified dysregulations showed predictive effect for drug response and prognosis as a whole (Supplementary Table S3, Supplementary Fig. S2). At last, 18 dysregulations that are robustly associated with prognosis were selected to build a prognostic signature, which proved to exhibit much higher predictive accuracy than mutation burden and neoantigen burden (Supplementary Fig. S3). Furthermore, the 18 dysregulations offer insightful clues to understand the mechanisms underlying prognosis, which endows the predictive signature with mechanistic explanatory power.

## Funding

This work was supported by the grants from National Key R&D Program of China [2018YFC0910500], National Natural Science Foundation of China [81672736], Shanghai Municipal Science and Technology [2017SHZDZX01 and 18DZ2294200] and NIH CPTAC (Cancer Proteomic Tumor Analysis Consortium) program.

*Conflict of Interest*: none declared.

## Supplementary Material

btaa688_Supplementary_Data
