# Supplementary material for: DysRegSig: an R package for identifying gene dysregulations and building mechanistic signatures in cancer
Source: Bioinformatics. 2020 Jul 27;37(3):429–30. doi: 10.1093/bioinformatics/btaa688 (PMC8058765; doi:10.1093/bioinformatics/btaa688)
Supplement: btaa688_Supplementary_Data [file btaa688_supplementary_data.zip › DysRegSig_supp_20200719.docx]

**Supplementary data**

DysRegSig: an R package for identifying gene dysregulations and building mechanistic signature in cancer

Quanxue Li^1,2§^, Wentao Dai^2, 3, 5§^, Jixiang Liu^2, 5^, Qingqing Sang^3^, Yi‑Xue Li^1,2, 4, 5^* and Yuan‑Yuan Li^2, 5^*

^1^ School of Biotechnology, East China University of Science and Technology, Shanghai 200237, China.

^2^ Shanghai Center for Bioinformation Technology, Shanghai 201203, China.

^3^ Department of Surgery, Shanghai Key Laboratory of Gastric Neoplasms, Shanghai Institute of Digestive Surgery, Ruijin Hospital, Shanghai Jiao Tong University School of Medicine, Shanghai, China

^4^ CAS Key Laboratory of Computational Biology, CAS-MPG Partner Institute for Computational Biology, Shanghai Institutes for Biological Sciences, Chinese Academy of Sciences, Shanghai 200031, China.

^5^ Shanghai Engineering Research Center of Pharmaceutical Translation & Shanghai Industrial Technology Institute, Shanghai Industrial Technology Institute, Shanghai 201203, China.

^§^These authors contributed equally to this work.

*To whom correspondence should be addressed.

**Contents**

[1 The design of DysRegSig 2](#_Toc41647110)

[1.1 Identify gene dysregulations 2](#_Toc41647111)

[1.2 Rank dysregulations and TFs 4](#_Toc41647112)

[1.3 Identify gene dysregulations that are robustly associated with a special phenotype for building predictive signature 5](#_Toc41647113)

[1.4 Related methods 6](#_Toc41647114)

[2 Case study 6](#_Toc41647115)

[2.1 Dataset 6](#_Toc41647116)

[2.2 Identify gene dysregulations with DysRegSig 7](#_Toc41647117)

[2.3 Rank TFs with dysregulation degree 7](#_Toc41647118)

[2.4 Check the predictive effect of dysregulations for drug response and prognosis 9](#_Toc41647119)

[2.5 Identify gene dysregulations that are robustly associated with prognosis 11](#_Toc41647120)

[Reference 13](#_Toc41647121)

## 1 The design of DysRegSig

DysRegSig is built for surveying gene dysregulations, that is, identifying the dysregulated gene regulatory relationships between two comparative conditions, from high-dimensional expression data with cooperativity and synergy between regulators and several other transcriptional regulation rules considered. The input of DysRegSig includes transcriptomic data corresponding to two conditions and a reference gene regulation network (GRN). DysRegSig includes one main module designed for identifying gene dysregulations, and two follow-up modules for ranking dysregulations and relevant TFs and screnning gene dysregulations that have robust contribution to a special phenotype for building predictive signature. The framework of DysRegSig is summarized in Figure 1a.

### 1.1 Identify gene dysregulations

DysRegSig identifies gene dysregulations by performing the following three steps.

#### 1.1.1 Step 1: Construct conditional GRNs

TF and the ‘best’ motif data were accessed from HumanTF database (Lambert, et al., 2018). 1000 bp upstream sequence from transcription start site of all coding genes were acquired by biomaRt and regarded as promoter regions (Durinck, et al., 2009). The promoter region of each gene was scanned with FIMO (Grant, et al., 2011) with p-value less than 1e-04. For each TF, the threshold that the probability of making at least one false discovery was less than 0.01 was adopted to perform multi-hypothesis test correction to select its targets. If a TF had more than 5000 targets, only the top 5000 targets ranked by FIMO score were kept. The remaining TF-target relationships, or candidate TF-targets, composed the reference GRN.

Based on expression data under specific condition, TF-target relationships in reference GRN are filtered by using feature selection algorithm. In DysRegSig package, we offer feature selection methods that is able to consider the cooperativity and synergy between TFs and robustly cope with high-dimensional data. The first method is *Boruta*, a wrapper around random forest algorithm (Kursa and Rudnicki, 2010). For each target, the expression values of its candidate TFs in reference GRN were regarded as original features, and the target’s expression was regarded as response variable. By shuffling candidate TFs, *Boruta* generated shadow features, iteratively estimated the importance of every TFs, and removed the TFs less relevant to its target. In this way, those conceptual links which did not perform functions under specific condition were removed from reference GRN, and the remaining TF-target relationships formed conditional GRNs. Besides, one other method that is able to consider the cooperativity and synergy between TFs and robustly cope with high-dimensional data, RGBM (Mall, et al., 2018), is also included in DysRegSig.

#### 1.1.2 Step 2: Measure regulatory intensity

We adopted linear regression to measure the regulatory intensity. The regression coefficients were taken as the regulatory intensities of regulation relationships, and their confidence intervals represented the estimated ranges. Supposing that there are k regulators of a target *T* in referece GRN, let the expression of *T* be ***Y****_n×1_* and the expression of regulators be ***X****_n×k_*, among them n means size of samples. Supposing that the expression data follows normal distribution, the general linear regression can be expressed as:

$\boldsymbol{Y}=\boldsymbol{X*b+}\boldsymbol{\varepsilon}\boldsymbol{,}\boldsymbol{\varepsilon}\boldsymbol{\sim N(0,}\boldsymbol{\sigma}^{\boldsymbol{2}}\boldsymbol{)}$ (1)

***ε*** means residual error and satisfies *Var*(***ε***) = $\boldsymbol{\sigma}^{\boldsymbol{2}}\boldsymbol{I}_{nn}$. In the classic setting, this model needs to satisfy n >> k. Ordinary least square yields the expectation and covariance of parameters as follows, which could be used to compare the difference in regulatory intensity of a specific regulation across conditions:

$\boldsymbol{b}_{\boldsymbol{o}}={\boldsymbol{(}\boldsymbol{X}^{\boldsymbol{T}}\boldsymbol{X)}}^{\boldsymbol{-1}}\boldsymbol{X}^{\boldsymbol{T}}\boldsymbol{Y}$ (2)

$Cov\left( \boldsymbol{b}_{\boldsymbol{o}} \right)=\boldsymbol{\sigma}^{\boldsymbol{2}}\boldsymbol{(X'X)}^{\boldsymbol{-1}}$ (3)

In order to overcome the obstacle that the matrix ***X^T^X*** is rank deficient when k > n in high-dimensional data, least absolute shrinkage and selection operator (LASSO) could be adopted to implement linear regression, which promotes sparse reconstructions through an *l_1_* penalty (Tibshirani, 1996). The estimation of parameters can be yielded as:

$\boldsymbol{b}_{\boldsymbol{l}}\left( \boldsymbol{Y,X;}\lambda\right)= arg\min_{b \in R^{k}} \left\{ \frac{1}{2n}\left\| \boldsymbol{Y-Xb} \right\|_{2}^{2}+\lambda\left\| \boldsymbol{b} \right\|_{1} \right\}$ (4)

In **formula (4)**, *λ* is generated from cross-validation. The limitation of canonical LASSO is that it could not generate confidence intervals of regression coefficients. To calculate the confidence intervals of regression coefficients for high-demensional data, we chose a de-basised LASSO method, which added a term proportional to a subgradient of the *l_1_* norm at the canonical LASSO solution $\boldsymbol{b}_{\boldsymbol{l}}$ to compensate the bias in the canonical LASSO(Javanmard and Montanari, 2014). The regression coefficients in de-biased LASSO method could be estimated as:

$\boldsymbol{b}_{\boldsymbol{u}}\left( \boldsymbol{Y,X;M,}\lambda\right)= \boldsymbol{b}_{\boldsymbol{l}}\left( \lambda\right)\boldsymbol{+}\frac{\boldsymbol{1}}{\boldsymbol{n}}\boldsymbol{M}\boldsymbol{X}^{\boldsymbol{T}}\boldsymbol{(Y-X*}\boldsymbol{b}_{\boldsymbol{l}}\left( \lambda\right)\boldsymbol{)}$ (5)

in which, $\boldsymbol{M}=\left( \boldsymbol{m}_{\boldsymbol{1}}\boldsymbol{,\ldots,}\boldsymbol{m}_{\boldsymbol{k}} \right)^{\boldsymbol{T}}$. Let ***m_i_*** be a solution of the convex program:

$$\mathrm{minimize}\boldsymbol{m}^{\boldsymbol{T}}\boldsymbol{\Sigma m}$$

$$subject to \left\| \boldsymbol{\Sigma}\boldsymbol{m}- \boldsymbol{e}_{\boldsymbol{i}} \right\|_{\infty}\leq\mu$$

$\mathrm{where}\boldsymbol{\Sigma}=\frac{\left( \boldsymbol{X}^{\boldsymbol{T}}\boldsymbol{X} \right)}{n}$, *e_i_*∈R^p^ is the vector with one at the *i*-th position and zero everywhere else; *μ* is the infinity-norm constraint on ***M***. According to the paper (Javanmard and Montanari, 2014), if any of the above problems is not feasible, then set ***M*** = I_p×p_.

From the derivation process of (5) in paper (Javanmard and Montanari, 2014), The covariance of $\boldsymbol{b}_{\boldsymbol{u}}$ can be expressed as:

$Cov\left( \boldsymbol{b}_{\boldsymbol{u}} \right)=\frac{\boldsymbol{\sigma}^{\boldsymbol{2}}\left( \boldsymbol{M\Sigma}\boldsymbol{M}^{\boldsymbol{T}} \right)}{n}$ (6)

$\boldsymbol{b}_{\boldsymbol{u}}$ has been approved as an approximately Gaussian distribution (Javanmard and Montanari, 2014). *1-α* confidence intervals of regression coefficients can be expressed as:

$\left[ \boldsymbol{b}_{\boldsymbol{u}}- \Phi\left( 1-\frac{\alpha}{2} \right)\boldsymbol{\sigma}\frac{\left| \boldsymbol{M\Sigma}\boldsymbol{M}^{\boldsymbol{T}} \right|_{i,i}^{\frac{1}{2}}}{\sqrt{n}}, \boldsymbol{b}_{\boldsymbol{u}}+\Phi\left( 1-\frac{\alpha}{2} \right)\boldsymbol{\sigma}\frac{\left| \boldsymbol{M\Sigma}\boldsymbol{M}^{\boldsymbol{T}} \right|_{i,i}^{\frac{1}{2}}}{\sqrt{n}} \right]$ (7)

In **formula (7)**, *Φ*(x) denotes cumulative distribution function of standard normal distribution. Regulatory intensity and its 95% confidence interval of every regulation under a certain condition could be estimated with **formula (5)** and **(7)**.

#### 1.1.3 Step 3: Identify gene dysregulations

We defined gene dysregulation by integrating three factors relevant to regulation, including regulatory intensity change, target expression change and the regulator’s contribution to the target.

For regulatory intensity change, we checked whether regulatory intensity of each regulation between different conditions was significantly different. Towards each regulation, if 95%, or other defined by user, confidence intervals of the regression coefficients have no overlap between normal and cancer conditions, the regulatory intensity could be regarded as significantly differential.

For target expression change, differential expression analysis was carried out. Differentially expressed genes (DEGs) were identified and their change directions, say activation of inhibition, were recorded.

For the regulator’s contribution to the target, since a target is always regulated by multiple regulators, the regulators whose regulatory intensity change were consistent with the target’s expression change from one condition to another were considered to play key roles in controlling targets expression.

At last, we identified gene dysregulations by adopting three standards. First, the regulatory intensity should be significantly different between conditions; secondly, target should be differentially expressed; lastly, regulatory intensity change should be consistent with the target’s expression change between conditions.

### 1.2 Rank dysregulations and TFs

The dysregulation degree of a dysregulation is measured by combing difference of regulatory intensities and target expression change between conditions. The dysregulation degree of a dysregulation (*Dysreg.DR*) can be expressed as:

$Dysreg.DR=\left( b_{u_{i,2}}-b_{u_{i,1}} \right)*{logFC}_{i}$ (8)

in which, $b_{u_{i,1}}$ and $b_{u_{i,2}}$ mean regulatory intensities in two conditions, and ${logFC}_{i}$ means target expression change.

The dysregulation degree of a TF is measured by the sum of dysregulation degree of all its dysregulations. The dysregulation degree of a TF (*Dysreg.TF*) can be expressed as:

$Dysreg.TF=\sum_{i=1}^{m} \left( b_{u_{i,2}}-b_{u_{i,1}} \right)*{logFC}_{i}$ (9)

Based on the quantification of *Dysreg.DR* and *Dysreg.TF*, DysRegSig offers tools to rank regulations and TFs, which could help researchers to focus on the most interesting genes and regulations.

### 1.3 Identify gene dysregulations that are robustly associated with a special phenotype for building predictive signature

We have tested that the identified gene dysregulations possess good prognostic effect in cancer (submitted for publication). DysRegSig offers a module for constructing mechanistic signature by combining dysregulations with using genetic algorithm. Genetic algorithm includes following steps: generation of initial population, fitness of evaluation, selection, crossover, and mutation (Scrucca, 2013).

Initial population: A population is composed of a number of individuals, which is also called as ‘chromosome’. ‘Gene’ is located along the ‘chromosome’, and the corresponding string position is called loci. In this work, each dysregulation is treated as one ‘gene’ in ‘chromosome’, and the length of ‘chromosome’ was the input number of dysregulations. Each ‘chromosome’ is encoded in binary way, in which 1 presents the appearance of a ‘gene’ and 0 presents the deficiency of a ‘gene’. The initial population with 1000 individuals is randomly generated.

Fitness: For prognosis, cross-validation C-index is used to evaluate the fitness of individuals. For each individual, the dysregulations appeared in its ‘chromosome’ are selected. The expression data of genes within the selected dysregulations is used to fit a cox model in training set, which is generated by randomly selecting a part of the samples. The remaining samples are used as test set to calculate the C-index. The cross-validation is repeated 10 times. Mean of theses C-indexes is returned as the fitness. For other objective, such as drug response, cross-validation AUC of classifying model could be used in the similar way as C-Index in prognosis. Besides, users could build their own fitness functions and use our framework to choose the best combination of dysregulations.

Selection: Based on the fitness, the individuals among top 20% are selected as the fitted group. Besides, 10% of individuals in the remaining individuals are randomly selected. The two parts are combined as the selected population.

Crossover and Mutation: The selected individuals reproduce and pass their ‘chromosome’ information to their offsprings. Two individuals are randomly picked out every time, and their ‘chromosomes’ are hybridized to generate offspring. The intersection rate is randomly selected between 0.1 and 0.9. Then mutation process occurs in offsprings. 10% of ‘genes’ among each individual are randomly mutated. The offspring whose ‘genes’ number is greater than 0 is reserved as survival offspring. Eventually, the offspring with same number as the initial population are generated.

The evolution process is iterated n (such as n=100) times. Users could select the “individual” with best performance of fitness function from the output of genetic algorithm and get the combination of dysregulations. To avoid random effect in genetic algorithm, we also offer a way to robustly select the important dysregulations for fitness function. For example, select the top 10 optimal individuals in each iteration, and calculate the occurring probability of each dysregulation in these individuals. the dysregulations that stably in top 10 individuals, which are supposed as important dysregulations for fitness function, are picked out at last.

### 1.4 Related methods

Fisher's z-test, which could test significant differences between two Pearson correlations, is popular to be used to implement differential correlation analysis (DCA) between two conditions in expression data (Fukushima, 2013). Several state-of-the-art approaches for DCA are built on this method (Fukushima, 2013; McKenzie, et al., 2016; Siska, et al., 2016). In this work, Pearson correlation coefficient for each TF-target in reference GRN was calculated in two different conditions. Then Fisher's z-test was used to test significant differences between two Pearson correlations. P values were adjusted by BH method (Benjamini and Hochberg, 1995). The results were treated as the output of DiffCor (differential correlation).

We furtherly combined two factors proposed in DysRegSig, target expression change and the regulator’s contribution to the target with DiffCor to generated a new method named as DiffCor++. In DiffCor++, differential gene expression analysis and the regulator’s contribution to the target were implemented in the same way as DysRegSig. Both DiffCor and DiffCor++ are also included in DysRegSig package.

For the rank of gene regulations in this study, the output of DiffCor was ranked by Fisher's z-value, the output of DiffCor++ was ranked by combining Fisher's z-value and target expression change between conditions in similar way as **formula (8)**.

## 2 Case study

### 2.1 Dataset

A dataset containing expression data and drug response data of PD-L1 inhibitor were derived from package IMvigor210CoreBiologies (Mariathasan, et al., 2018). The gene expression is quantified by read counts, which was transformed with voom to log2-counts per million with associated precision weights (Law, et al., 2014). The values less than 0 were treated as missing values in mRNA expression dataset. For a certain gene, when the number of missing values was larger than 20% of total sample size, the gene was deleted. The remaining missing data was filled in with kNN method (Troyanskaya, et al., 2001). The log2-transformd expression matrix was used for downstream analysis.

### 2.2 Identify gene dysregulations with DysRegSig

Gene dysregulation analysis was carried out between response group and no-response group. The drug response level of each sample to each treatment was labelled with complete response (CR), partial response (PR), stable disease (SD) and progressive disease (PD). In this work, CR (n=25) and PR (n=43) were categorized into response group, SD (n=63) and PD (n=167) was categorized into no-response group. Gene dysregulations between response group and no-response group were extracted based on the criteria: the 90% confidence intervals of the regression coefficient have no overlap between normal and cancer conditions, target is differential expression gene filtered by p-value < 0.05 with limma (Smyth, 2005), regulatory intensity change is consistent with the target’s expression change between conditions. At last, 295 gene dysregulations were identified (Table S1). Over-representation pathways were extracted in ConcensusPathDB with the threshold that pathway had at least 5 genes overlapped with input genes and p-value was less than 0.05 (Herwig, et al., 2016). A series of immune response or cancer related pathways were enriched, such as Toll-like receptor cascade, B cell receptor signaling pathway, NFAT-dependent transcription in lymphocytes, TGF-beta signaling pathway, cell cycle, apoptosis, EGF-EGFR signaling pathway, VEGF-VEGFR signaling pathway, Notch signaling pathway, PI3K-Akt signaling pathway, TP53 signaling pathway, and so on.

### 2.3 Rank TFs with dysregulation degree

The dysregulation degree of TFs was quantified with Formula (9), and was used to rank TFs. The top 10 TFs were listed in Table S2. Most of these TFs have been reported to participate in key carcinogenic processes, excepting ZNF776 and ZNF71. Among them, MBNL2 was reported to be a key regulator for mediating pre-mRNA alternative splicing and participating in controlling the pluripotency of embryonic stem cells (Han, et al., 2013), and also taken as a TF in humanTF database (Lambert, et al., 2018). In our results, several gene dysregulations are linked to MBNL2 (Table S1), which offers useful clues for exploring the roles of MBNL2 in transcriptional regulation during carcinogenesis. In summary, the results indicates the feasibility and effectivity of our gene dysregulation analysis tool for exploring the underlying dysrefuncitonal mechanisms of cancers.

Table S2. The top ranked TFs based on dysregulation degree.

| **Rank** | **TF** | **Dysregulation degree** | **Main functions in cancer** | **PMID** |
| --- | --- | --- | --- | --- |
| 1 | NFIB | 0.71156707 | Promote metastasis | 27374332, 27373156 |
| 2 | NR5A2 | 0.61844767 | Link to differentiation and inflammation | 29443959 |
| 3 | FOXL1 | 0.57805595 | Induce apoptosis | 23801748 |
| 4 | ZNF776 | 0.55455355 | —— | —— |
| 5 | KLF7 | 0.53648879 | Regulate cell proliferation | 22936656, 29716672 |
| 6 | SP4 | 0.53398448 | Promote angiogenesis | 17409437, 16788159 |
| 7 | MBNL2 | 0.51885103 | Inhibit metastasis | 31320607 |
| 8 | CTCF | 0.51389981 | Regulate chromatin conformation and gene expression | 19563753, 30086769 |
| 9 | ZNF71 | 0.50102497 | —— | —— |
| 10 | GATA6 | 0.49561885 | Regulate epithelial differentiation | 18536717, 27325420 |

It was also noticed that NFIB ranked top in our dysregulation list. NFIB is a member of nuclear factor I (NFI) transcription factors, and participates in diverse processes including cell differentiation, cell proliferation, cell metastasis, and EMT (Chen, et al., 2017; Denny, et al., 2016). Four dysregulations were identified to be linked to NFIB, NFIB-MT1E, NFIB-TICRR, NFIB-DUSP7, NFIB-AP3M2. MT1E is a metallothionein, its expression is positively correlated with cancer cell migration and tumor stage (Wu, et al., 2008). In no-response group, NFIB has weak effect on expression of MT1B; while in response group, NFIB inhibits expression of MT1B (Figure S1a). Our dysregulation analysis offers a path to connect NFIB and MT1E in terms of metastasis.

TICRR is a regulator of DNA replication and S/M and G2/M checkpoints (Boos, et al., 2013). Whether TICRR accelerates or stalls fork extension remains controversial (Maya-Mendoza, et al., 2018; Sansam, et al., 2015; Yu, et al., 2019). Dysregulated DNA replication and abrogation of checkpoint response to DNA stress in cancer force cells to enter mitosis with incompletely replicated DNA, thus leading to accumulation of DNA damage. It is well-known that high level of DNA damage is related to better response of immune checkpoint inhibitors. It is speculated that TICRR is related to response of atezolizumab. In our results, NFIB promotes expression of TICRR in response group, while weakly regulates TICRR expression in no-response group (Figure S1b). Additionally, the higher expression of TICRR is related to better prognosis (HR= 0.837, 95%CI: 0.7405~0.946, p-value=0.004). These results might support the positive effect of TICRR on immunotherapy. Consider the close relation of TICRR with DNA damage, the connection between TICRR and response of immunotherapy is worth in-depth study.

DUSP7 is a mitogen-activated protein kinase (MAPK) phosphatase (MKP) that act as negative regulators of MAPK activity (Keyse, 2008). NFIB has weak regulatory effect on DUSP7 in no-response group, but shows strong inhibition effect in response group in our result (Figure S1c). AP3M2 is a subunit of the heterotetrameric adaptor-related protein complex 3 (AP-3), which plays a role in protein trafficking to lysosomes and specialized organelles (Robinson, 2004). Similar to the dysregulation NFIB-DUSP7, NFIB has weak regulatory effect on AP3M2 in no-response group, but shows strong inhibition effect in response group (Figure S1d). Given that DUSP7 and AP3M2 participate key processes in manimal cells, it is worthy to furtherly study their function in imunotheray, and our gene dysregulation results might offer some clues.


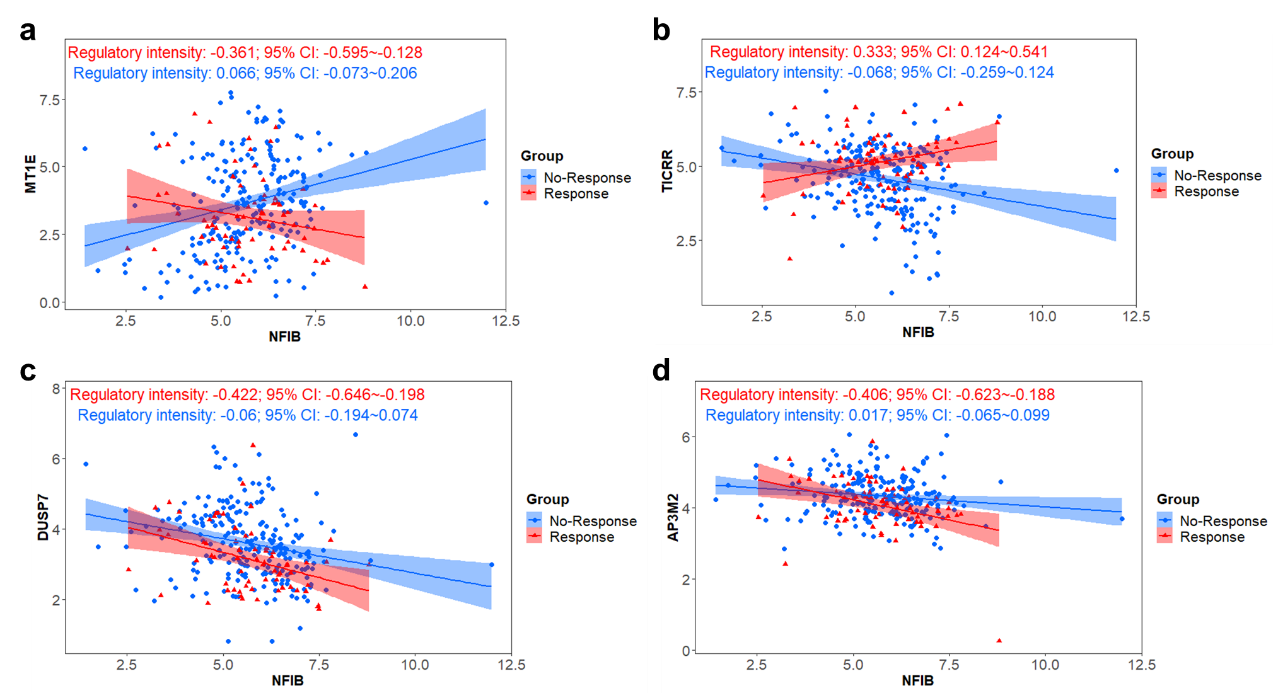


Figure S1. The expression pattern of dysregulations related to NFIB between two conditions. (a) NFIB-MT1E. (b) NFIB-TICRR. (c) NFIB-DUSP7. (d) NFIB-AP3M2. X-axis denotes TF’s expression level and y-axis denotes target’s expression level. One point corresponds to one sample, with red representing response group and blue representing no-response group. The regression lines and confidence interval shadows were calculated by single variable regression and used to visualize the differences of gene regulation between conditions.

### 2.4 Check the predictive effect of dysregulations for drug response and prognosis

We then checked the predictive effect of the identified dysregulations for drug response and prognosis. For drug response, every two genes involved in each gene dysregulation identified by DysRegSig were used to build SVM models for predicting drug response, and the predictive accuracy was measured by AUC. For prognosis, every two genes involved in each gene dysregulation identified by DysRegSig were used to fit cox models for overall survival (OS), and the predictive accuracy was measured by C-Index. The values of AUC or C-Index of every gene dysregulations were compared with seven types of control settings as follows, Compare_1: two genes were randomly selected from the gene list of processed expression data; Compare_2: one differentially expressed gene (DEG) and one non-differentially expressed gene (non-DEG) were randomly selected from the gene list of processed expression data; Compare_3: two DEGs were randomly selected; Compare_4: one regulation was randomly selected from the reference GRN; Compare_5: one regulation whose target is DEG was randomly selected from the reference GRN; Compare_6: one gene pair was randomly selected from the output of DiffCor (with threshold p.value < 0.01); Compare_7: one regulation was randomly selected from the output of DiffCor++ (with threshold p.value < 0.01). For each type of control, the same number of random gene pairs as dysregulations identified by DysRegSig were generated. The SVM model was constructed for drug response prediction and the cox model was fitted for OS prediction with each random pair, and the accuracy indexes AUC and C-Index were then calculated respectively. At last, for each type of control, Wilcox test was used to check whether the AUCs or C-Indexes from the identified dysregulations were significantly larger than those from random pairs (one-way Wilcox test), that is, whether the accuracy of SVM models or cox models built by dysregulations as a whole was higher than controls. This process was repeated 100 times.

The results of predictive effect of the 295 DysRegSig-identified dysregulations for drug response and prognosis were presented in Table S3 and Figure S2. For drug response, AUCs of 295 SVM models built with the 295 dysregulations were significantly larger than controls in Compare_1 and Compare_4, with the median p.value of 100 times of Wilcox tests less than 0.05, and also larger than controls in Compare_2, Compare_5, and Compare_6, with the median p.value of 100 times of Wilcox tests less than 0.1. For prognosis, C-Indexs of 295 models fitted with the 295 dysregulations were significantly larger than controls in Compare_1, Compare_4, and Compare_6, with the median p.value of 100 times of Wilcox tests less than 0.05. Taken together, except Compare_7 for drug response and Compare_3 for prognosis, the median p.value of 100 times of Wilcox tests were less than 0.5, which indicate that the dysregulations identified by DysRegSig have predictive effect for drug response and prognosis as a whole.

Table S3. The predictive effect of the identified dysregulations for drug response and prognosis. Data are expressed as median (first quantile ~ third quantile) p value of 100 times of Wilcox tests.

| **Compare** | **Drug response** | **Prognosis** |
| --- | --- | --- |
| Compare_1 | 0.0128(0.0048~0.0402) | 0.0038(0.0011~0.0187) |
| Compare_2 | 0.0829(0.0379~0.2121) | 0.1128(0.0377~0.1860) |
| Compare_3 | 0.3557(0.2350~0.5268) | 0.5807(0.4675~0.7106) |
| Compare_4 | 0.0069(0.0018~ 0.0296) | 0.0377(0.0077~0.0791) |
| Compare_5 | 0.0976(0.0625~ 0.1796) | 0.3513(0.1755~0.5659) |
| Compare_6 | 0.0753(0.0171~ 0.1544) | 0.0357(0.0106~0.0790) |
| Compare_7 | 0.5388(0.3622~ 0.6714) | 0.1204(0.0391~0.2323) |


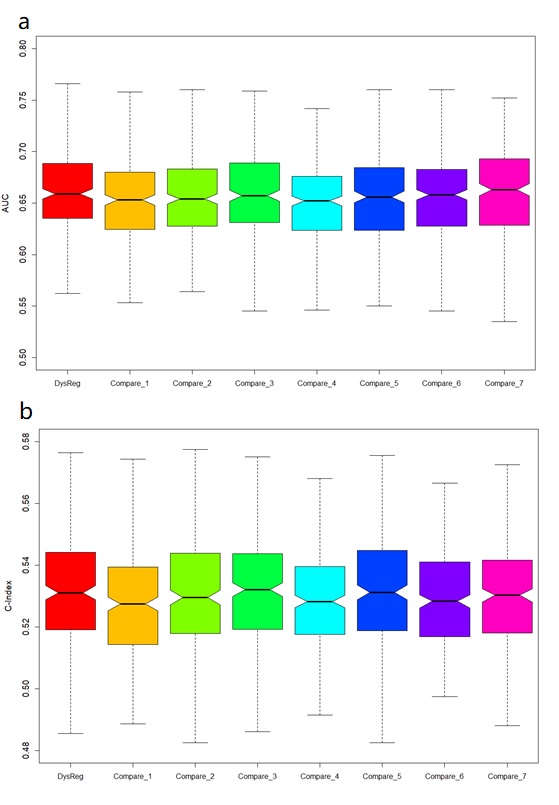


Figure S2. The boxplot of predictive effect of the identified dysregulations. (a) The AUC of DysReg and the seven comparisons for drug response. (b) The C-Index between DysReg and the seven comparisons for prognosis.

### 2.5 Identify gene dysregulations that are robustly associated with prognosis

Driven by that the dysregulations identified by DysRegSig show good predictive effect for drug response and prognosis as a whole in this case study, we expect to identify gene dysregulations that are robustly associated to prognosis and build high-accuracy mechanistic signature with dysregulations. There is a general consensus among both clinicians and biologists about the need for signatures with mechanistic interpretability as well as high predictive accuracy for cancer precision medicine (Jia, et al., 2009; Lu, et al., 2019; Robinson, et al., 2013; Topalian, et al., 2016). It could also be expected that taking mechanistic interpretation into consideration would further enhance the predictive accuracy and robustness of signatures in clinical application (Robinson, et al., 2013). As a mechanism-driven strategy-based method, DysRegSig has the potential to provide functionally relevant seeds for building predictive signatures with both predictive power and explanatory power for cancer prognosis.

DysRegSig includes a module designed for screening the gene dysregulations that are robustly associated to a special phenotype with genetic algorithm. At here, a group of gene dysregulations that have robust association with OS were obtained and used to buid a prognostic signature. Based on the rank of gene dysregulations based on formula (8), the top 100 dysregulations were conducted to the module. In order to avoid random effect in genetic algorithm, we select the top 10 optimal “individuals”, and calculate the occurring probability of each dysregulation in these “individuals”. After 100 iterations in genetic algorithm, 18 dysregulations are stably maintained in top 10 “individuals”, including POU5F1-HIST1H3G, ZNF331-REEP6, ZNF596-FBN2, NFIB-MT1E, KLF8-FHOD3, PBX3-KIF20A, ZNF486-SLAIN1, NR3C1-EVA1C, EGR1-DCLK2, HOXA10-PDIK1L, ZNF382-COL16A1, ZNF180-LTBP2, MSC-NHSL2, USF2-ADORA2B, ZNF619-ZDHHC13, KLF12-FCGRT, ZNF816-CASP6 (Figure S3a and S3b). The total 36 genes among these 18 dysregulations were used to build a prognostic signature for OS, without using any other information. Time-dependent ROC showed that AUC at 0.5-, 1-, and 1.5- year OS reached 0.74, 0.79 and 0.82 in the dataset (Figure S3c) (Blanche, et al., 2013), which were much higher predictive accuracy than that of mutation burden and neoantigen burden (Figure S3d and S3e).The cross-validation results also showed the higher accuracy of dysregulation based signature (Table S4). Furthermore, the dysregulational information contained in dysregulations offers a path to to understand the mechanisms for prognosis and give explanatory power to the signature. This case states that our new gene dysregulation analysis tool, DysRegSig, could help to build prognostic signature with high accuracy as well as explanatory power.

Table S4. Comparing prognostic accuracy of Dysreg_signature built in this case to FMOne mutation burden per MB and Neoantigen burden with cross-validation in terms of C-Index, AUC of time-dependent ROC at 0.5-, 1-, and 1.5- year survival in IMvigor210CoreBiologies dataset. In cross-validation, 60% of the samples were randomly selected as training set to construct a cox model, and the left 40% of samples were taken as testing set to calculate C-Index, AUC of time-dependent ROC at 0.5-, 1-, and 1.5- year survival of the cox model. Data are expressed as median (first quantile-third quantile) of 100 times of cross-validation results.

| Indicator | Dysreg_signature | FMOne mutation burden per MB | Neoantigen burden per MB |
| --- | --- | --- | --- |
| C-Index | 0.641(0.621-0.660) | 0.598(0.576-0.610) | 0.582(0.567-0.599) |
| AUC_0.5 | 0.664(0.632-0.695) | 0.600(0.574-0.624) | 0.589(0.554=0.611) |
| AUC_1.0 | 0.701(0.667-0.722) | 0.644(0.622-0.665) | 0.657(0.626-0.684) |
| AUC_1.5 | 0.701(0.675-0.732) | 0.643(0.608-0.670) | 0.677(0.644-0.719) |


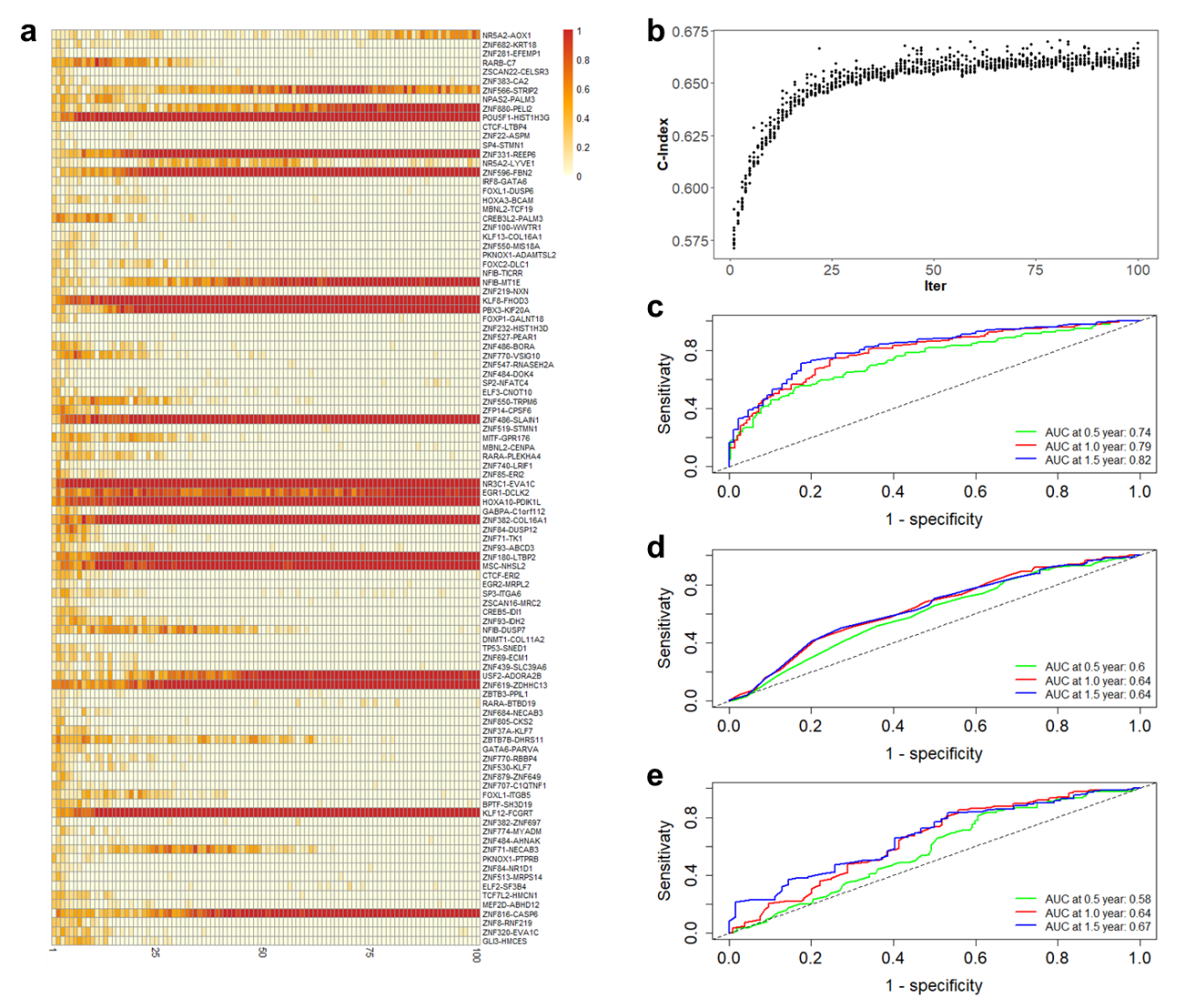


Figure S3. The prognostic signature built with gene dysregulations. (a) The frequency of each dysregulation in top ten “individuals” at each iteration of genetic algorithm. The color means the frequency (0~1). (b) The boosting process of top ten C-Indexes during iteration. (c) Time-dependent ROC for OS based on the 18 dysregulations (36 genes) prognostic signature. (d) Time-dependent ROC for based on FMOne mutation burden per MB. (e) Time-dependent ROC for OS based on Neoantigen burden per MB.

## Reference

Benjamini, Y. and Hochberg, Y. Controlling the False Discovery Rate: A Practical and Powerful Approach to Multiple Testing. *Journal of the Royal Statistical Society: Series B (Methodological)* 1995;57(1):289-300.

Blanche, P., Dartigues, J.F. and Jacqmin-Gadda, H. Estimating and comparing time-dependent areas under receiver operating characteristic curves for censored event times with competing risks. *Statistics in medicine* 2013;32(30):5381-5397.

Boos, D., Yekezare, M. and Diffley, J.F. Identification of a heteromeric complex that promotes DNA replication origin firing in human cells. *Science* 2013;340(6135):981-984.

Durinck, S.*, et al.* Mapping identifiers for the integration of genomic datasets with the R/Bioconductor package biomaRt. *Nat Protoc* 2009;4(8):1184-1191.

Fukushima, A. DiffCorr: an R package to analyze and visualize differential correlations in biological networks. *Gene* 2013;518(1):209-214.

Grant, C.E., Bailey, T.L. and Noble, W.S. FIMO: scanning for occurrences of a given motif. *Bioinformatics* 2011;27(7):1017-1018.

Herwig, R.*, et al.* Analyzing and interpreting genome data at the network level with ConsensusPathDB. *Nat Protoc* 2016;11(10):1889-1907.

Javanmard, A. and Montanari, A. Confidence intervals and hypothesis testing for high-dimensional regression. *Journal of Machine Learning Research* 2014;15(1):2869-2909.

Jia, J.*, et al.* Mechanisms of drug combinations: interaction and network perspectives. *Nature reviews. Drug discovery* 2009;8(2):111-128.

Keyse, S.M. Dual-specificity MAP kinase phosphatases (MKPs) and cancer. *Cancer metastasis reviews* 2008;27(2):253-261.

Kursa, M.B. and Rudnicki, W.R. Feature Selection with the Boruta Package. *Journal of statistical software* 2010;36(11):13.

Lambert, S.A.*, et al.* The Human Transcription Factors. *Cell* 2018;172(4):650-665.

Law, C.W.*, et al.* voom: Precision weights unlock linear model analysis tools for RNA-seq read counts. *Genome Biol* 2014;15(2):R29.

Lu, Y.*, et al.* Dynamic edge-based biomarker non-invasively predicts hepatocellular carcinoma with hepatitis B virus infection for individual patients based on blood testing. *Journal of molecular cell biology* 2019;11(8):665-677.

Mall, R.*, et al.* RGBM: regularized gradient boosting machines for identification of the transcriptional regulators of discrete glioma subtypes. *Nucleic Acids Res* 2018;46(7):e39.

Mariathasan, S.*, et al.* TGFbeta attenuates tumour response to PD-L1 blockade by contributing to exclusion of T cells. *Nature* 2018;554(7693):544-548.

Maya-Mendoza, A.*, et al.* High speed of fork progression induces DNA replication stress and genomic instability. *Nature* 2018;559(7713):279-284.

McKenzie, A.T.*, et al.* DGCA: A comprehensive R package for Differential Gene Correlation Analysis. *BMC Syst Biol* 2016;10(1):106.

Robinson, M.S. Adaptable adaptors for coated vesicles. *Trends Cell Biol* 2004;14(4):167-174.

Robinson, W.H.*, et al.* Mechanistic biomarkers for clinical decision making in rheumatic diseases. *Nat Rev Rheumatol* 2013;9(5):267-276.

Sansam, C.G.*, et al.* Cyclin-dependent kinase regulates the length of S phase through TICRR/TRESLIN phosphorylation. *Genes & development* 2015;29(5):555-566.

Scrucca, L. GA: A Package for Genetic Algorithms in R. *J Stat Softw* 2013;53(4):1-37.

Siska, C., Bowler, R. and Kechris, K. The discordant method: a novel approach for differential correlation. *Bioinformatics* 2016;32(5):690-696.

Smyth, G.K. limma: Linear Models for Microarray Data. 2005.

Tibshirani, R. Regression Shrinkage and Selection Via the Lasso. *Journal of the Royal Statistical Society: Series B (Methodological)* 1996;58(1):267-288.

Topalian, S.L.*, et al.* Mechanism-driven biomarkers to guide immune checkpoint blockade in cancer therapy. *Nature reviews. Cancer* 2016;16(5):275-287.

Wu, Y.*, et al.* Overlapping gene expression profiles of cell migration and tumor invasion in human bladder cancer identify metallothionein 1E and nicotinamide N-methyltransferase as novel regulators of cell migration. *Oncogene* 2008;27(52):6679-6689.

Yu, Q.*, et al.* TICRR Contributes to Tumorigenesis Through Accelerating DNA Replication in Cancers. *Front Oncol* 2019;9:516.
